# MiR-323b-5p acts as a novel diagnostic biomarker for critical limb ischemia in type 2 diabetic patients

**DOI:** 10.1038/s41598-018-33310-4

**Published:** 2018-10-10

**Authors:** Biao Cheng, Ju-yi Li, Xiao-chao Li, Xiu-fang Wang, Zhong-jing Wang, Jue Liu, Ai-ping Deng

**Affiliations:** 1 Department of Pharmacy, The Central Hospital of Wuhan, Tongji Medical College, Huazhong University of Science and Technology, Wuhan, 430021 Hubei China; 2 Department of Research, Wuhan Hospital of Traditional Chinese and Western Medicine, Huazhong University of Science and Technology, Wuhan, 430000 Hubei China; 3 Department of Pain, The Central Hospital of Wuhan, Tongji Medical College, Huazhong University of Science and Technology, Wuhan, 430021 Hubei China; 4 Department of Endocrinology, The Central Hospital of Wuhan, Tongji Medical College, Huazhong University of Science and Technology, Wuhan, 430021 Hubei China

## Abstract

Type 2 diabetes mellitus (T2DM) is a major contributor to peripheral artery disease (PAD), especially in cases that advance to critical limb ischemia (CLI). Accumulating evidence indicates that miRNAs play an important role in the development of PAD and T2DM. Due to the limited value of current diagnostic methods for CLI in T2DM patients, we compared the miRNA expression profiles of Chinese T2DM patients with or without CLI to find out whether distinctive miRNAs could serve as potential diagnostic biomarkers. We statistically identified 7 miRNAs (hsa-miR-200b-3p, hsa-miR-2115-3p, hsa-miR-431-5p, hsa-miR-486-5p, hsa-miR-210-3p, hsa-miR-1264, hsa-miR-323b-5p) which were up-regulated in the CLI group, whereas other 4 miRNAs (hsa-miR-5579-3p, hsa-miR-665, hsa-miR-4285, hsa-miR-500a-3p) were down-regulated. Our validation test suggested a relatively high diagnostic accuracy of serum hsa-miR-323b-5p levels for the detection of CLI in T2DM patients, with a sensitivity of 62.67% and a specificity of 80.65%. The area under the curve (AUC) for miR-323b-5p + confounding risk factors was 0.94 (95% CI: 0.884–0.994, *P* < 0.001), which was higher than that for miR-323b-5p. Taken together, our results indicate that circulating hsa-miR-323b-5p could be a promising serum biomarker for the diagnosis of critical limb ischemia in type 2 diabetic patients.

## Introduction

Peripheral artery disease (PAD) is a progressive atherosclerotic disorder that affected almost 155 million people worldwide in 2015^1^. With progressive aging of the global population, the prevalence of PAD in the ≥75 year-old age group has increased from 12.5% in 2003 to 18.5% in 2012^2^, resulting in increased risks of hospitalization and mortality^3,4^. Critical limb ischemia (CLI) is the most severe stage of PAD, and can lead to ischemic rest pain, tissue ulcers, gangrene and death^5,6^. In fact, more than 60% of CLI patients will be dead 5 years after diagnosis^7^.

Type 2 diabetes mellitus (T2DM) is a major contributor to CLI^8^. It has been reported that the risk of major cardiovascular events increases by 26% for each 1% increase in HbA1c levels^9^. Epidemiological studies have revealed that diabetic patients are fivefold more likely to develop CLI than non-diabetic patients^10,11^, while the mortality rate is more than 3 times higher due to the high incidence of cardiovascular disease events^12–14^. It has been suggested that the main mechanisms underlying the formation of lower limb emboli in diabetic patients involve oxidative stress, persistent inflammation and damaged endothelial tissues^15–17^. However, the exact mechanisms remain unclear.

Moreover, studies conducted in Germany revealed that approximately 2.7–4% of symptomatic PAD patients go on to develop CLI after 1-year^18^ and more than 40% of these CLI patients will suffer lower limb amputations^19,20^; 70% of these patients are diabetic^18^. Therefore, it is necessary to diagnose, predict and treat CLI at an early stage in PAD patients in order to decrease the limb loss and mortality rates. Currently, several methods are used to diagnose CLI, such as the ankle-brachial index (ABI), color Doppler ultrasound examination, and transcutaneous oxygen pressure^19,21,22^. However, these tests are only partially useful in diabetics with PAD. For example, researchers have shown that the risk factors associated with a reduced ABI can vary and are different in patients with or without diabetes^23^; thus, the diagnostic value of ABI is limited^24^. Therefore, investigation of novel biomarkers for early diagnosis and prognosis, as well as more effective therapies, would benefit diabetic patients with PAD.

MicroRNAs (miRNAs) are small, non-coding RNA molecules (containing about 22 nucleotides) that regulate gene expression and mediate complex biological processes^25^. It has been reported that multiple pathologic conditions, including CVD^26^, DM^27^, cancers^28^ and PAD^29^, are associated with altered miRNAs. These molecules are being studied to better understand the pathophysiology of many diseases, and as potential biomarkers for diagnosis^30,31^. Previous studies have shown that the expression of several miRNAs, such as let 7e, miR-27b, miR-130a and miR-210, is significantly different between patients with lower limb atherosclerosis and age-matched controls^29,32,33^. However, the miRNA profile of diabetic patients with PAD is still unclear, especially when CLI is present. Therefore, in the present study we investigated whether the expression of miRNAs in T2DM patients with or without CLI was significantly different and evaluated the diagnostic value of miRNAs for the diagnosis of CLI in patients with T2DM.

## Materials and Methods

### Subjects

A total of 66 T2DM patients were recruited from the Central Hospital of Wuhan from May 1^st^ 2015 to May 1^th^ 2017, including 31 subjects with CLI (CLI group) and 35 subjects without CLI (non-CLI group). Exclusion criteria included type 1 diabetes, cancer, severe liver or kidney failure, and receiving therapy for any chronic inflammatory disease. All participants were Han Chinese and thus of the same genetic background. This study was approved by the Ethics Committee of the Central Hospital of Wuhan and was conducted in accordance with the principles of the Declaration of Helsinki. All samples were collected after obtaining a written informed consent from the participants.

T2DM was diagnosed according to current American Diabetes Association criteria^34^. Briefly, T2DM was defined as fasting glucose levels ≥17.0 mmol/L, or a 2-h plasma glucose value ≥ 11.1 mmol/L during an oral glucose tolerance test or glycated hemoglobin levels >6.5%. CLI was diagnosed based on ABI < 0.9 and lower extremity arterial steno is >50% by color Doppler ultrasound examination^35,36^. Patients with ABI values equal to or greater than 0.9 and lower than 1.3, and with a normal lower extremity arterial pressure were defined as non-CLI.

### Plasma collection and RNA extraction

Peripheral whole blood (5 mL) was collected in ethylenediaminetetraacetic acid (EDTA) tubes and stored at −80 °C until analysis. RNA was extracted from plasma using a miRNeasy serum/plasma kit (TIANGEN: catalog number DP503, China), according to the manufacturer’s instructions. RNA quality was determined by means of an Agilent 2100 Bioanalyzer (Agilent Technologies, Santa Clara, USA). RNA was quantified using a Nanodrop 2000 Spectrophotometer (Thermo Scientific, Wilmington, USA).

### miRNAs microarray hybridization

RNA samples were obtained from T2DM patients with CLI (n = 4) or without CLI (n = 4), matched for age, gender, and diabetes duration. The isolated total RNA from each subject was labeled using the miRCURY^TM^ Power labeling kit (Exiqon, Inc., Vedbaek, Denmark) and hybridized to the miRCURY^TM^ Array v16.0, according to the standard protocol. Hybridized arrays were analyzed by means of the GenePix 4000B microarray scanner (Molecular Devices, LLC., Sunnyvale, CA, USA), and the images were digitized with the Array-Pro image analysis software (Media Cybernetics, Silver Spring, MD, USA). Raw fluorescence data were processed using GenePix Pro 6.0 software (Molecular Devices, LLC., Sunnyvale, CA, USA). Individual spot fluorescence data were normalized based on the background and positive control fluorescence intensities.

### Quantitative reverse transcription polymerase chain reaction (qRT-PCR)

Quantification of miRNAs was performed through a two-step reaction process: reverse transcription (RT) and PCR. cDNA synthesis was carried out by using a reverse transcriptase kit (TIANGEN; catalog number: KR211, China). miRNAs were quantified using SYBR Green (TIANGEN; catalog number FP411, China), as previously described^37^. The qRT-PCR reactions were carried out by heating at 95 °Cfor 1 min, followed by 40 cycles of 95 °C for 10 sec, 60 °C for 30 sec, and 70 °C for 10 sec. Duplicate assays were performed for each sample.

### Target Prediction and Gene Ontology (GO) Analysis

Potential miRNA targets were predicted using the TargetScan human 7.0^38^ (http://targetscan.org) and mirDB^39^ (http://www.mirdb.org/) web servers, and overlapping results from the two servers were considered the final predicted targets. Gene Ontology (GO) and KEGG (Kyoto Encyclopedia of Genes and Genomes) pathway analyses were conducted using the DAVID (Database for Annotation, Visualization and Integrated Discovery) bioinformatics resource.

### Statistics

Statistical analysis was performed using SPSS (version 18.0) and GraphPad Prism (version 5.0) software. Continuous variables were expressed as the mean ± SEM or as the median (interquartile range), and categorical variables were compared by means of Fisher’s exact test or the χ^2^ test. The independent t-test or Fisher-Pitman Permutation test were used to compare normally distributed data, while the non-parametric Mann-Whitney U-test or Exact Mann-Whitney rank sum test was used to compare data with a non-normal distribution. *P* values were calculated using the Benjamini and Hochberg method to correct for multiple testing. Volcano plots (log fold-change versus negative log (base 10) *P*-values) were used to display the different biomarker distributions between CLI patients and non-CLI controls. Binary logistic regression analysis was performed to determine independent predictors of CLI in T2DM patients. All correlations were analyzed using the Pearson method or nonparametric Spearman method depending on the distribution of the data. Receiver operating characteristic (ROC) curves were used to evaluate the potential diagnostic value of each miRNA based on its ability to discriminate between T2DM patients with or without CLI. *P* < 0.05 was considered statistically significant.

## Results

### Clinical Characteristics

The study consisted of two groups: T2DM patients with CLI (n = 35), and T2DM patients without CLI (n = 31). The clinical data of all the study subjects and of the miRNA array cohort group are compared in Table 1. T2DM duration, hypertension, smoking, ABI, FPG, HbA1c, TC and Cr parameters were significantly different between T2DM patients with or without CLI (*P* < 0.05, exact *P* value shown in Table 1). Specifically in the miRNA array cohort group, there were significant differences in terms of ABI and TC (*P* = 0.007 and 0.048, respectively), but no differences were detected in other parameters (*P* > 0.05, exact *P* value shown in Table 1).

**Table 1:** General characteristic of the study subjects.

	miRNA array cohort		*P*	validation cohor		*P*
	CLI	Non - CLI		CLI	Non - CLI	
No. of subjects	4	4	—	27	31	—
Age, y	65.00 ± 1.73	65.50 ± 1.55	0.837	64.30 ± 2.73	61.0, (56.0–69.0)	0.790^‡^
T2D Duration, y	10.00 ± 1.41	12.25 ± 1.80	0.363	15.48 ± 1.47	9.0, (8.0–14.0)	*0.004* ^‡^
Women, %	2, (50.00)	2, (50.00)	1.000	14, (51.85)	16, (51.61)	0.986^†^
Hyperlipidemia, %	0, (0)	2, (50.00)	0.429	10, (37.04)	10, (32.26)	0.702^†^
Hypertension, %	2, (50.00)	2, (50.00)	1.000	16, (59.26)	9, (29.03)	*0.020* ^†^
Smoking, %	2, (50.00)	2, (50.00)	1.000	15, (55.56)	8, (25.81)	*0.021* ^†^
ABI	0.46 ± 0.10	1.06 ± 0.03	*0.007*	24.10 (22.70–25.80)	1.11 ± 0.01	*0.000* ^‡^
BMI, kg/m^2^	23.80 ± 1.21	25.98 ± 1.99	0.386	24.95 ± 0.57	25.46 ± 0.52	0.512
CAD, %	2, (50.00)	2, (50.00)	1.000	8, (29.63)	8, (25.81)	0.745^†^
Statin use, %	0, (0)	1, (25.00)	1.000	6, (22.22)	4, (12.90)	0.349^†^
Antihypertensive treatment use, %	2, (50.00)	1, (25.00)	1.000	8, (29.63)	5, (16.13)	0.219^†^
FPG, mmol/L	8.27 ± 2.69	7.87 ± 1.52	0.899	9.60 (8.65–14.36)	7.27 (6.14–9.05)	*0.001* ^‡^
HbA1c, %	10.35 ± 1.15	7.48 ± 0.31	0.509	8.90 (7.90–9.60)	6.90 (6.50–9.00)	*0.001* ^‡^
HDL-C, mmol/L	0.97 ± 0.08	1.06 ± 0.06	0.372	1.08 ± 0.05	1.16 (0.90–1.32)	0.281^‡^
LDL-C, mmol/L	2.14 ± 0.16	3.10 ± 0.41	0.100	2.63 ± 0.17	2.63 ± 0.15	0.987
TG, mmol/L	1.29 ± 0.33	1.37 ± 0.45	0.901	1.48 (1.07–1.86)	1.29 (0.92–1.84)	0.847^‡^
TC, mmol/L	3.51 ± 0.23	4.69 ± 0.42	*0.048*	2.80 (2.55–3.68)	4.30 ± 0.18	*0.006* ^‡^
Apo A, g/L	1.09 ± 0.07	1.19 ± 0.04	0.248	1.16 (1.03–1.29)	1.15 (1.06–1.35)	0.172^‡^
Apo B, g/L	0.80 ± 0.06	0.96 ± 0.10	0.194	0.92 ± 0.04	0.86 ± 0.04	0.265
Cr, μmol/L	55.50 ± 6.36	72.75 ± 9.58	0.184	75.00 (58.00–107.00)	65.00 (55.00–72.00)	*0.044* ^‡^
BUN, mmol/L	4.95 ± 0.93	6.24 ± 1.06	0.393	5.60 (4.60–7.24)	6.11 ± 0.40	0.374^‡^
UA, mmol/L	268.00 ± 10.96	262.25 ± 39.80	0.897	327.97 ± 13.45	323.76 ± 18.77	0.860
Total bilirubin, mol/L	11.63 ± 1.48	10.03 ± 1.71	0.586	12.51 ± 0.97	12.50 ± 0.87	0.995
hsCRP, mg/dL	1.24 ± 0.69	0.64 ± 0.25	0.446	0.35 (0.19–1.36)	0.64 (0.26–2.30)	0.402^‡^
ALT, U/L	18.00 ± 7.53	22.00 ± 5.02	0.674	14.60 (8.00–26.00)	14.00 (10.90–23.00)	0.343^‡^
AST, U/L	21.75 ± 3.78	22.50 ± 2.72	0.877	19.00 (13.00–31.00)	19.28 ± 1.25	0.136^‡^

ABI, Ankle brachial index; BMI, body mass index; CAD, coronary artery disease; FPG, fasting plasma glucose; HbA1c, glycosylated hemoglobin; HDL-C, high density lipoprotein cholesterol; LDL-C, low density lipoprotein cholesterol; TG, triglyceride; TC, total cholesterol; Apo A, Apolipoprotein A; Apo B, Apolipoprotein B; Cr, serum creatinine; BUN, blood urea nitrogen; UA, uric acid; hsCRP, high-sensitivity C-reactive protein; ALT, alanine aminotransferase; AST, aspartate aminotransferase. Data is expressed as mean ± SEM, median (interquartile range) or percentage. Significant values are marked in italic. Continuous variales were compared by the Fisher-Pitman Permutation test, and categorical variables were compared by Fisher’s exact test in the miRNA array cohort, however, the validation cohort was analyzed using ^†^χ^2^ test or ^‡^Mann-Whitney U-test, and the others were analyzed using t-tests.

### Identification of different miRNA profiles in T2DM patients with and without CLI

**Figure 1:**
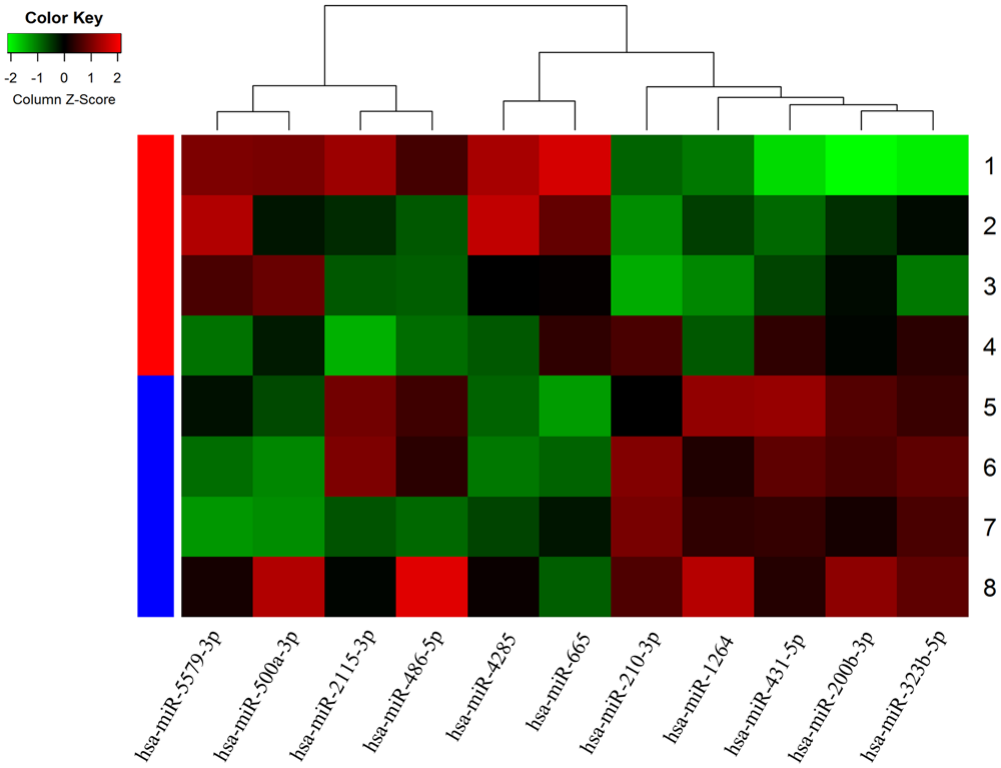
Hierarchical cluster analysis of 4 T2DM patients with CLI and 4 T2DM patients without CLI, based on differentially expressed miRNAs. Red indicates relative up-regulation, and green indicates relative down-regulation.

To identify miRNAs that were differentially expressed between the two groups, we employed miRNA microarrays. The serum levels of eleven miRNAs were significantly different between diabetes mellitus patients with CLI (n = 4) and without CLI (n = 4) (Fig. 1). Briefly, 7 miRNAs, including hsa-miR-200b-3p, hsa-miR-2115-3p, hsa-miR-431-5p, hsa-miR-486-5p, hsa-miR-210-3p, hsa-miR-1264, hsa-miR-323b-5p were up-regulated in the CLI group (mean ratio = 1.57–4.18, *P* < 0.05; exact *P* value shown in Table 2), whereas 4 other miRNAs, including hsa-miR-5579-3p, hsa-miR-665, hsa-miR-4285, hsa-miR-500a-3p were down-regulated (mean ratio = 0.04–0.53, *P* < 0.05; exact *P* value shown in Table 2).

**Table 2:** Differentially expression miRNAs identified by microarray analysis in plasma between T2DM with CLI and without CLI controls.

Differentially expression type	microRNA name	Mean ratio	*P* value
Up-regulated	hsa-miR-200b-3p	2.6211	0.016
	hsa-miR-2115-3p	1.8945	0.040
	hsa-miR-431-5p	4.1855	0.042
	hsa-miR-486-5p	1.5782	0.029
	hsa-miR-210-3p	3.7626	0.036
	hsa-miR-1264	2.3110	0.011
	hsa-miR-323b-5p	2.0134	0.034
Down-regulated	hsa-miR-5579-3p	0.0474	0.038
	hsa-miR-665	0.3571	0.026
	hsa-miR-4285	0.5322	0.044
	hsa-miR-500a-3p	0.2741	0.044

### Validation of the miRNA microarray results by quantitative RT-PCR

Based on the microarray results and on their recently reported function, two miRNAs were selected for further validation: (1) miR-323b-5p, which is a newly described miRNA. Scarce information exists about this miRNA in terms of disease prediction, although it has been reported to be associated with an increased risk of gestational diabetes mellitus in pregnant Chinese Han women^40^; (2) miR-500a-3p, which is known to be significantly down-regulated in the plasma of patients with advanced atherosclerotic lesions^41^ and is also down-regulated in diabetic cardiomyopathy in the Akita mouse model^42^.

**Figure 2:**
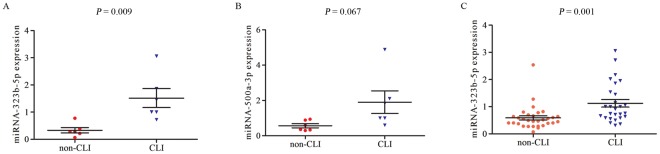
Validation of miRNAs expression by qRT-PCR. Initial assessment of (**A**) miRNA-323b-5p and (**B**) miRNA-500a-3p expression in six CLI and six non-CLI patients. (**C**) Validation of miRNA-323b-5p expression in a larger number of CLI (n = 27) and non-CLI (n = 31) patients. The p-values were calculated by means of the Mann-Whitney U-test. CLI: T2DM patient with CLI, non-CLI: T2DM patient without CLI.

We initially tested the expression levels of these two miRNAs in a small number of diabetic subjects with CLI (n = 6) and without CLI (n = 6). The results showed that the expression of miR-323b-5p was significantly upregulated in the CLI group when compared with the non-CLI group (*P* = 0.009, Fig. 2A). In contrast, there was no significant difference in miR-500a-3p expression between the two groups (*P* = 0.067, Fig. 2B). Therefore, we tested the expression levels of miR-323b-5p in a larger number of subjects (n = 27 in the CLI group and n = 31 in the non-CLI group). The results showed that miR-323b-5p expression was significantly higher (1.9 fold) in T2DM patients with CLI than in T2DM patients without CLI (*P* = 0.001, Fig. 2C).

### Association between miR-323b-5p levels and baseline clinical parameters with T2DM and CLI

In order to investigate the association between miR-323b-5p levels and CLI in relation to baseline factors, binary logistic regression analysis was conducted.

After adjusting for T2D duration, hypertension, smoking, FPG, HbA1c, TC, Cr, statins treatment and antihypertensive treatment, increased miR-323b-5p levels were found to be associated with an increased odds ratio (OR) (OR = 8.170) for T2DM with CLI (*P* = 0.032) (Table 3).

**Table 3:** Binary logistic regression analysis for the risk factors of CLI in T2DM patients.

	OR	95% CI for OR	*P*	OR^*^	95% CI for OR^*^	*P* ^*^
T2D Duration	1.173	1.044–1.318	0.007	∕	∕	∕
Hypertension	3.556	1.194–10.588	0.032	∕	∕	∕
Smoking	3.594	1.189–10.862	0.023	∕	∕	∕
FPG	1.360	1.108–1.668	0.003	∕	∕	∕
HbA1c	1.765	1.185–2.628	0.005	∕	∕	∕
TC	0.528	0.324–0.861	0.010	∕	∕	∕
Cr	1.019	1.000–1.038	0.048	∕	∕	∕
Statin treatment	0.519	0.129–2.077	0.519	∕	∕	∕
Antihypertensive treatment	0.457	0.129–1.617	0.224	∕	∕	∕
miRNA-323b-5p	6.756	1.644–27.770	0.008	8.170	1.029–64.864	0.032

FPG, fasting plasma glucose; HbA1c, glycosylated hemoglobin; TC, total cholesterol; Cr, serum creatinine. All study subjects were included in the analysis. Significant values are marked in italic.

### Correlation between miR-323b-5p levels and clinical parameters

Next, we examined the correlation between miR-323b-5p levels and clinical parameters, such as T2D duration, hypertension, smoking, FPG, HbA1c, TC, Cr, statin treatment and antihypertensive treatment in T2DM patients with CLI (Table 4). Our results indicate that miR-323b-5p expression levels were positively correlated with smoking (r = 0.275, *P* = 0.037), FPG (r = 0.285, *P* = 0.030) and HbA1c (r = 0.335, *P* = 0.010). However, no significant correlation was found between the levels of miR-323b-5p and T2D duration, TC, Cr, statins treatment or antihypertensive treatment (*P* > 0.05, exact *P* value shown in Table 4).

**Table 4:** Spearman Rho correlations between plasma miRNA-323b-5p level and CLI risk factors in T2DM patients.

Variable		miRNA-323b-5p
T2D Duration	*rho*	0.251
	*p*	0.059
Hypertension	*rho*	0.201
	*p*	0.131
Smoking	*rho*	0.275
	*p*	*0.037*
FPG	*rho*	0.285
	*p*	*0.030*
HbA1c	*rho*	0.335
	*P*	*0.010*
TC	*rho*	−0.255
	*p*	0.053
Cr	*rho*	0.055
	*p*	0.682
Statin use	*rho*	−0.205
	*p*	0.124
Antihypertensive treatment	*rho*	−0.079
	*p*	0.555

CI, confidence interval; FPG, fasting plasma glucose; HbA1c, glycosylated hemoglobin; TC, total cholesterol; Cr, serum creatinine. *Adjusted for T2D Duration, hypertension, smoking, FPG, HbA1c, TC, Cr. All study subjects were included in the analysis. Significant values are marked in italic.

### miR-323b-5p: Target prediction, function and pathway analysis

**Figure 3:**
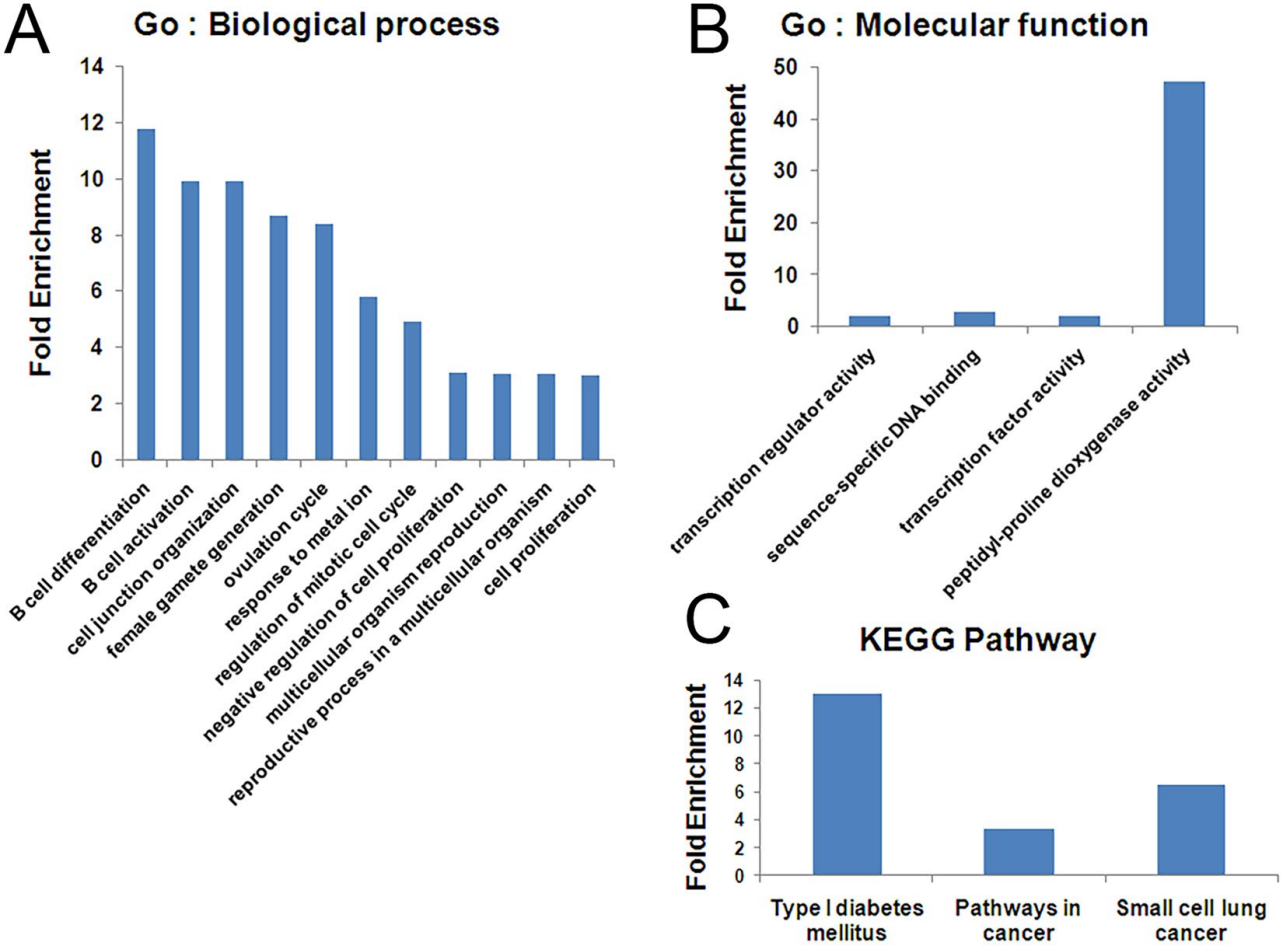
Functional analysis of miRNA-323b-5p targets in T2DM patients with CLI. (**A**) Significantly enriched GO: biological process terms. (**B**) GO: molecular function terms. (**C**) KEGG pathways (*p* value < 0.05).

To investigate the possible biological function of miR-323b-5p, we conducted GO and KEGG pathway analyses using the DAVID online analysis tool. Eleven GO: biological process terms (GO: bp), 4 GO: molecular function terms (GO: mf) and 3 KEGG pathways were identified (Fig. 3). The most significantly enriched GO: bp terms were “GO: 0030183~B cell differentiation”, “GO: 0042113~B cell activation” and “GO: 0034330~cell junction organization” (Fig. 3A). The most enriched GO: mf term was “GO: 0031543~peptidyl-proline dioxygenase activity” (Fig. 3B). On the other hand, two significantly enriched KEGG pathways were identified, including hsa04940: type I diabetes mellitus and hsa05200: pathways in cancer (*P* < 0.05) (Fig. 3C). The exact GO and KEGG pathway analysis information, including GO IDs, pathway IDs, *P* values, fold enrichments and target genes, is shown in Tables S1 and S2.

In addition, we predicted miR-323b-5p target genes by means of TargetScan and mirDB software. There were 105 and 2244 predicted target genes by Targetscan and miRDB, respectively. Only 92 genes overlapped between the two types of software (Table S3).

### Evaluation of miR-323b-5p for the diagnosis of T2DM patients with CLI

**Figure 4:**
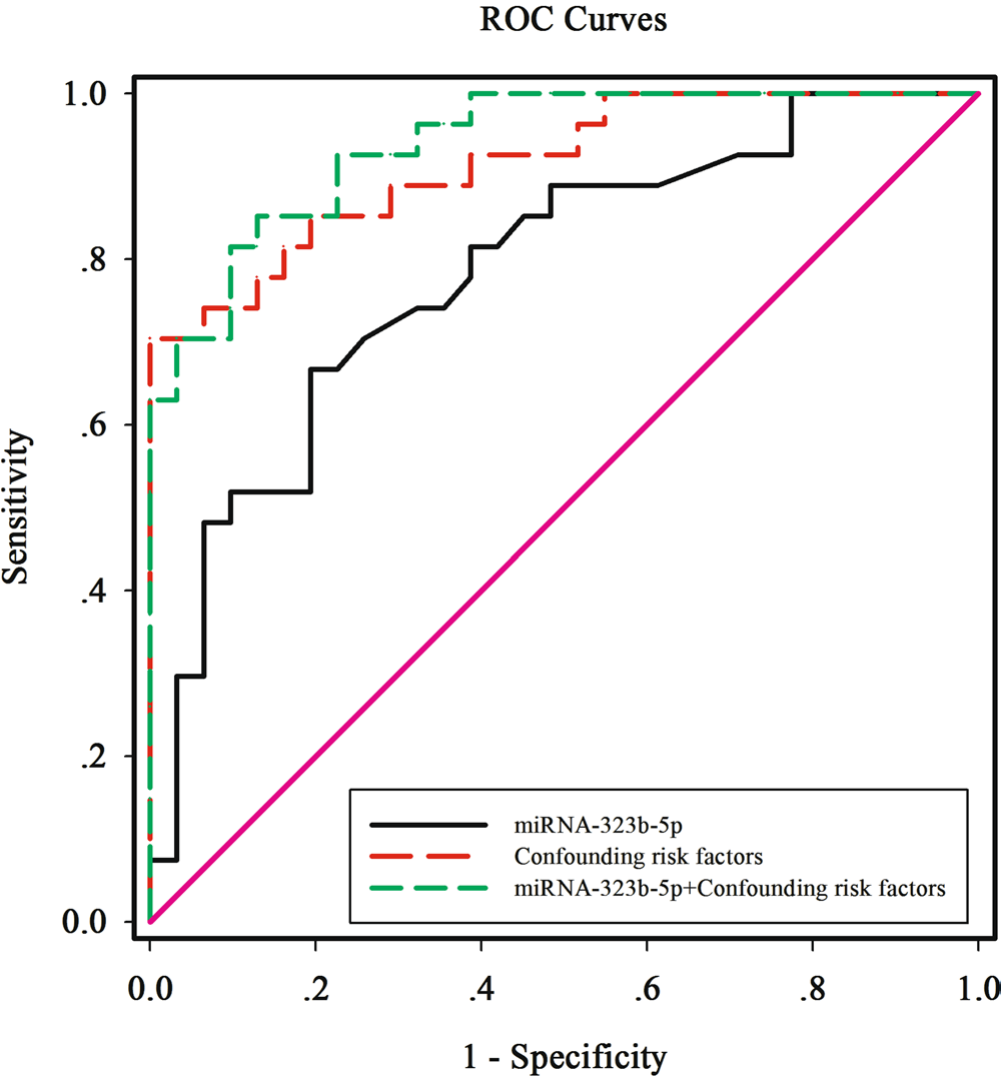
ROC curve analysis of the miRNA-323b-5p cut off point for the presence of CLI in T2DM patients. The AUC for miRNA-323b-5p was 0.78 (*p* < 0.001), the identified miR-323b-5p cut off point was 0.71, the Youden index was 0.47, the sensitivity was 62.67% and the specificity was 80.65%. The AUC for miRNA-323b-5p + confounding risk factors was 0.94 (*p* < 0.001). The confounding risk factors included T2DM duration, hypertension, smoking, FPG, HbA1c, TC, Cr, stain treatment and antihypertensive treatment. FPG, fasting plasma glucose; HbA1c, glycosylated hemoglobin; TC, total cholesterol; Cr, serum creatinine.

We explored the potential value of miR-323b-5p as a biomarker for the diagnosis of CLI in T2DM patients by means of (ROC) curve analysis. As shown in Fig. 4, the area under the curve (AUC) for miR-323b-5p was 0.78 (95% confidence intervals (CI): 0.667–0.903, *P* < 0.001) and the optimal cut-off point for miR-323b-5p was 0.71. The ROC curve analysis indicated that miR-323b-5p showed a relatively high diagnostic accuracy for the diagnosis of CLI in T2DM patients, with a Youden index of 0.47, a sensitivity of 62.67% (95% Cl: 0.460–0.835) and a specificity of 80.65% (95% Cl: 0.625–0.926). The AUC for miR-323b-5p + confounding risk factors (including T2DM duration, hypertension, smoking, FPG, HbA1c, TC, Cr, statin treatment and antihypertensive treatment) was 0.94 (95% CI: 0.884–0.994, *P* < 0.001), which was higher than that for miR-323b-5p (95% CI: 0.024–0.0285, *P* = 0.021) and for confounding risk factors (95% CI: −0.066–0.114, *P* = 0.602) (Fig. 4).

## Discussion

It is widely believed that diabetes mellitus is a critical risk factor for PAD and death^43^, due to increased arterial calcification and endothelial dysfunction^44^. Studies have shown that calcification or reduced arterial wall elasticity can affect the accuracy of ABI measurements and lead to false results, especially in CLI patients^45,46^. For example, there were no significant differences in the ABI values of 269 Japanese CLI patients with or without diabetes^47^. Therefore, due to the limitations of current diagnostic methods for CLI in diabetes patients and the high risk of amputation, identification of accurate and sensitive biomarkers is of the utmost importance.

Increasing evidence suggests that miRNAs play a significant role in the diagnosis of T2DM or as therapeutic targets^48–50^ and may also regulate endothelial cell function and angiogenesis^51,52^. In this study, we found significant differences in the serum miRNA expression profiles of Han Chinese T2DM patients with or without CLI. Seven miRNAs were up-regulated and four miRNAs were down-regulated in T2DM patients with CLI (Fig. 1). ROC curve analysis indicated that one up-regulated miRNA, hsa-miR-323b-5p, could be a specific biomarker for CLI in T2DM (Fig. 4, *P* < 0.001).

In addition to diabetes, it is well established that in almost 70% of PAD patients there are additional risk factors, such as age, hypertension, dyslipidemia and smoking^53^, and this is consistent with our logistic regression analysis results. Our results showed significant differences between T2DM patients with and without CLI in terms of smoking, FPG, HbA1c, TC and Cr, miR-323b-5p (Table 3, *P* < 0.05). Additional correlation analysis results showed that the expression levels of miR-323b-5p were positively correlated with smoking (*P* = 0.037), FPG (*P* = 0.030) and HbA1c (*P* = 0.010) (Table 4). However, after adjusting for all risk factors including T2DM duration, hypertension, smoking, FPG, HbA1c, TC, Cr, statins treatments and antihypertensive treatment, miR-323b-5p remained a significant association with an increased odds ratio (OR) for diabetes with CLI. All these results suggest that miR-323b-5p is independently and strongly associated with T2DM patients with CLI.

MiR-323b-5p is a relatively new microRNA. So far, there are only a few studies investigating its functions in cell growth and proliferation^54,55^. However, various studies have shown that miRNAs which mediate cell differentiation and proliferation may also affect endothelial cell function and be associated with tissue ischemia^56^. For instance, miR let-7, which is aberrantly expressed in myocardial infarction, arrhythmia, angiogenesis, atherosclerosis, and hypertension^57^, has also been reported to inhibit Bcl-xl expression and to mediate ox-LDL-induced endothelial apoptosis through the regulation of cell proliferation, migration, autophagy and apoptosis^58^. Furthermore, Li and coworkers showed that a total of 84 miRNAs were differentially expressed between RAW 264.7 macrophages and foam cells induced by ox-LDL, and GO terms and KEGG pathways analyses revealed that the target genes of most of these miRNAs were enriched for cell differentiation^59^. Similarly, our GO and KEGG pathways analysis for miR-323b-5p showed significant enrichment in the biological processes of B cell differentiation, cell proliferation and apoptosis, suggesting the presence of common mechanisms with the miRNAs mentioned above. However, the biological function, bioinformatics analysis and target gene information reports are limited to miR-323b-5p in CLI patients with T2DM.

In the present study, we predicted 92 possible miR-323b-5p target genes in CLI patients with T2DM by means of TargetScan and mirDB software. However, only limited relevant information was found on these genes. A global gene expression dataset suggested that miR-323b-5p was mainly present in adipose tissue^60^. Stuart and coworkers showed that miR-323b-5p levels were increased in familial combined hyperlipidemia patients and that this miRNA could induce lipid accumulation in 3T3-L1 cells by reducing endogenous CDKN2B (cyclin-dependent kinase 4 2B) protein levels^61^, suggesting that CDKN2B may participate in adipose tissue metabolism. Furthermore, a recent study showed that CDKN2B RNA may indirectly regulate CAD-associated genes via targeting miR-92a^62^. Our results showed that CDKN2B was one of the 92 possible target genes of miR-323b-5p. Therefore, CDKN2B RNA could be a target gene of miR-323b-5p, although more studies would be needed to verify this assumption.

In summary, our study showed that eleven circulating miRNAs were significantly differentially expressed between T2DM patients with and without CLI. The validation study indicated that the diagnostic accuracy of serum hsa-miR-323b-5p was significantly higher than that of other miRNAs, suggesting that miR-323b-5p could be a potential biomarker for the diagnosis of CLI in diabetes patients. However, the exact role played by miR-323b-5p during the development of CLI is still unknown. Further investigations are required to clarify the underlying mechanisms as well as the biological functions of this miRNA.

## Electronic supplementary material

Supplementary Information
