# Supplementary material for: MiR-323b-5p acts as a novel diagnostic biomarker for critical limb ischemia in type 2 diabetic patients
Source: Sci Rep. 2018 Oct 10;8:15080. doi: 10.1038/s41598-018-33310-4 (PMC6179988; doi:10.1038/s41598-018-33310-4)
Supplement: Supplementary file 1 — Supplementary Information [file 41598_2018_33310_MOESM1_ESM.doc]

MiR-323b-5p acts as a novel diagnostic biomarker for critical limb ischemia in type 2 diabetic patients

Biao Cheng^1†^, Ju-yi Li^1†^, Xiao-chao Li^2^, Xiu-fang Wang^3^, Zhong-jing Wang^4^, Jue Liu^1*^, Ai-ping Deng^1*^

^1^ Department of Pharmacy, The Central Hospital of Wuhan, Tongji Medical College, Huazhong University of Science and Technology, Wuhan 430021, Hubei, China

^2^ Department of Research, Wuhan Hospital of Traditional Chinese and Western Medicine, Huazhong University of Science and Technology, Wuhan 430000, Hubei, China

^3^ Department of Pain, The Central Hospital of Wuhan, Tongji Medical College, Huazhong University of Science and Technology, Wuhan 430021, Hubei, China

^4^ Department of Endocrinology, The Central Hospital of Wuhan, Tongji Medical College, Huazhong University of Science and Technology, Wuhan 430021, Hubei, China

^†^Biao Cheng and Ju-yi Li contributed equally to this work.

^*^Correspondence and requests for materials should be addressed to Aiping Deng, Email: dapyxb@163.com, Tel: +862782201237; Jue Liu, Email: 15562399@qq.com, Tel: +862782201756.

**Table S1**. **General characteristic of the GO analysis.**

| **GO ID** | **Term** | ***P* value** | **Fold enrichment** | **Genes** |
| --- | --- | --- | --- | --- |
| **Biological process** | | | | |
| GO:0042113 | B cell activation | 0.007 | 9.88 | LRRC8A, BCL2, CEBPG, HSPD1 |
| GO:0032504 | multicellular organism reproduction | 0.013 | 3.08 | FMN2, NPAS3, ZMIZ1, BCL2, TSNAX, PEBP1, NHLH2, PCYT1B |
| GO:0048609 | reproductive process in a multicellular organism | 0.013 | 3.08 | FMN2, NPAS3, ZMIZ1, BCL2, TSNAX, PEBP1, NHLH2, PCYT1B |
| GO:0030183 | B cell differentiation | 0.026 | 11.74 | LRRC8A, BCL2, CEBPG |
| GO:0008283 | cell proliferation | 0.026 | 3.01 | COPS2, BCL2, CD274, HSPD1, MXD1, IL1A, NUMBL |
| GO:0010038 | response to metal ion | 0.030 | 5.82 | ALG2, BCL2, ALDOB, PEBP1 |
| GO:0034330 | cell junction organization | 0.036 | 9.88 | ITGA6, BCL2, NUMBL |
| GO:0008285 | negative regulation of cell proliferation | 0.040 | 3.12 | TMEM115, LEPRE1, CDKN2B, BCL2, CD274, IL1A |
| GO:0007346 | regulation of mitotic cell cycle | 0.045 | 4.94 | CDKN2B, BCL2, PEBP1, IL1A |
| GO:0007292 | female gamete generation | 0.045 | 8.67 | FMN2, ZMIZ1, BCL2 |
| GO:0042698 | ovulation cycle | 0.048 | 8.41 | BCL2, NHLH2, PCYT1B |
| **Cellular component** | | | | |
| GO:0005794 | Golgi apparatus | 0.021 | 2.36 | TMEM115, C6ORF25, ST3GAL6, RNF24, PEBP1, ST8SIA3, CLASP2, LMAN1, TRIP11, GGA2 |
| GO:0045202 | synapse | 0.026 | 3.48 | SVOP, MPDZ, GLRA2, SNTB1, PEBP1, SYT9 |
| GO:0044456 | synapse part | 0.029 | 4.19 | SVOP, MPDZ, GLRA2, PEBP1, SYT9 |
| GO:0030135 | coated vesicle | 0.040 | 5.18 | SVOP, PEBP1, SYT9, HSPD1 |
| **Molecular function** | | | | |
| GO:0030528 | transcription regulator activity | 0.004 | 2.11 | PLAG1, COPS2, BTAF1, TAF5, CEBPG, SPEN, MXD1, NPAS3, MEOX2, BCL2, TRPS1, PAX8, SMARCD1, HOMEZ, NHLH2, TRIP11, TCF12 |
| GO:0043565 | sequence-specific DNA binding | 0.013 | 2.78 | MEOX2, TAF5, TRPS1, CEBPG, PAX8, HOMEZ, TSNAX, HSPD1, TCF12 |
| GO:0003700 | transcription factor activity | 0.029 | 2.12 | PLAG1, BTAF1, MEOX2, TAF5, TRPS1, CEBPG, PAX8, HOMEZ, SPEN, MXD1, TCF12 |
| GO:0031543 | peptidyl-proline dioxygenase activity | 0.041 | 47.03 | LEPRE1, EGLN1 |

**Table S2**. **General characteristic of the KEGE pathway analysis.**

| **Pathway ID** | **Term** | ***P* value** | **Fold enrichment** | **Genes** |
| --- | --- | --- | --- | --- |
| hsa04940 | Type I diabetes mellitus | 0.020 | 12.97 | HLA-DPB1, HSPD1, IL1A |
| hsa05200 | Pathways in cancer | 0.026 | 3.32 | CDKN2B, ITGA6, BCL2, PAX8, JAK1, EGLN1 |
| hsa05222 | Small cell lung cancer | 0.072 | 6.48 | CDKN2B, ITGA6, BCL2 |

**Table S3**. **The 92 genes which are overlapped predicted by Targetscan and miRDB.**

| **No.** | **miRNA** | **Target gene** | **TargetScan7.1** | **mirdbV5** | **NumSum** |
| --- | --- | --- | --- | --- | --- |
| 1 | hsa-miR-323b-5p | IGLL5 | 1 | 1 | 2 |
| 2 | hsa-miR-323b-5p | RNF24 | 1 | 1 | 2 |
| 3 | hsa-miR-323b-5p | BOD1L2 | 1 | 1 | 2 |
| 4 | hsa-miR-323b-5p | IL1A | 1 | 1 | 2 |
| 5 | hsa-miR-323b-5p | PGRMC2 | 1 | 1 | 2 |
| 6 | hsa-miR-323b-5p | PEBP1 | 1 | 1 | 2 |
| 7 | hsa-miR-323b-5p | CALB1 | 1 | 1 | 2 |
| 8 | hsa-miR-323b-5p | ALDOB | 1 | 1 | 2 |
| 9 | hsa-miR-323b-5p | MEOX2 | 1 | 1 | 2 |
| 10 | hsa-miR-323b-5p | LEPRE1 | 1 | 1 | 2 |
| 11 | hsa-miR-323b-5p | TMEM115 | 1 | 1 | 2 |
| 12 | hsa-miR-323b-5p | STK38 | 1 | 1 | 2 |
| 13 | hsa-miR-323b-5p | SNTB1 | 1 | 1 | 2 |
| 14 | hsa-miR-323b-5p | LMAN1 | 1 | 1 | 2 |
| 15 | hsa-miR-323b-5p | EGLN1 | 1 | 1 | 2 |
| 16 | hsa-miR-323b-5p | JAK1 | 1 | 1 | 2 |
| 17 | hsa-miR-323b-5p | GATC | 1 | 1 | 2 |
| 18 | hsa-miR-323b-5p | RHOBTB2 | 1 | 1 | 2 |
| 19 | hsa-miR-323b-5p | SLC25A36 | 1 | 1 | 2 |
| 20 | hsa-miR-323b-5p | PCTP | 1 | 1 | 2 |
| 21 | hsa-miR-323b-5p | TSNAX | 1 | 1 | 2 |
| 22 | hsa-miR-323b-5p | FAM179B | 1 | 1 | 2 |
| 23 | hsa-miR-323b-5p | BTAF1 | 1 | 1 | 2 |
| 24 | hsa-miR-323b-5p | SYT9 | 1 | 1 | 2 |
| 25 | hsa-miR-323b-5p | CD274 | 1 | 1 | 2 |
| 26 | hsa-miR-323b-5p | KCNJ16 | 1 | 1 | 2 |
| 27 | hsa-miR-323b-5p | GLRA2 | 1 | 1 | 2 |
| 28 | hsa-miR-323b-5p | SERPINB2 | 1 | 1 | 2 |
| 29 | hsa-miR-323b-5p | PAX8 | 1 | 1 | 2 |
| 30 | hsa-miR-323b-5p | CDH12 | 1 | 1 | 2 |
| 31 | hsa-miR-323b-5p | SETD7 | 1 | 1 | 2 |
| 32 | hsa-miR-323b-5p | LRRC8A | 1 | 1 | 2 |
| 33 | hsa-miR-323b-5p | BCL2 | 1 | 1 | 2 |
| 34 | hsa-miR-323b-5p | GPC6 | 1 | 1 | 2 |
| 35 | hsa-miR-323b-5p | MCHR2 | 1 | 1 | 2 |
| 36 | hsa-miR-323b-5p | NUMBL | 1 | 1 | 2 |
| 37 | hsa-miR-323b-5p | PLAG1 | 1 | 1 | 2 |
| 38 | hsa-miR-323b-5p | ST8SIA3 | 1 | 1 | 2 |
| 39 | hsa-miR-323b-5p | NHLH2 | 1 | 1 | 2 |
| 40 | hsa-miR-323b-5p | HSPD1 | 1 | 1 | 2 |
| 41 | hsa-miR-323b-5p | PCYT1B | 1 | 1 | 2 |
| 42 | hsa-miR-323b-5p | TP53INP2 | 1 | 1 | 2 |
| 43 | hsa-miR-323b-5p | TUBG2 | 1 | 1 | 2 |
| 44 | hsa-miR-323b-5p | SAV1 | 1 | 1 | 2 |
| 45 | hsa-miR-323b-5p | MPDZ | 1 | 1 | 2 |
| 46 | hsa-miR-323b-5p | SVOP | 1 | 1 | 2 |
| 47 | hsa-miR-323b-5p | KIF5C | 1 | 1 | 2 |
| 48 | hsa-miR-323b-5p | PTBP2 | 1 | 1 | 2 |
| 49 | hsa-miR-323b-5p | TAF5 | 1 | 1 | 2 |
| 50 | hsa-miR-323b-5p | SH3RF3 | 1 | 1 | 2 |
| 51 | hsa-miR-323b-5p | C4orf19 | 1 | 1 | 2 |
| 52 | hsa-miR-323b-5p | DNAJB5 | 1 | 1 | 2 |
| 53 | hsa-miR-323b-5p | TCF12 | 1 | 1 | 2 |
| 54 | hsa-miR-323b-5p | PTPN4 | 1 | 1 | 2 |
| 55 | hsa-miR-323b-5p | NPAS3 | 1 | 1 | 2 |
| 56 | hsa-miR-323b-5p | CHP1 | 1 | 1 | 2 |
| 57 | hsa-miR-323b-5p | SMARCD1 | 1 | 1 | 2 |
| 58 | hsa-miR-323b-5p | FBXO34 | 1 | 1 | 2 |
| 59 | hsa-miR-323b-5p | ZMIZ1 | 1 | 1 | 2 |
| 60 | hsa-miR-323b-5p | UMODL1 | 1 | 1 | 2 |
| 61 | hsa-miR-323b-5p | TMEM231 | 1 | 1 | 2 |
| 62 | hsa-miR-323b-5p | PLP1 | 1 | 1 | 2 |
| 63 | hsa-miR-323b-5p | AK4 | 1 | 1 | 2 |
| 64 | hsa-miR-323b-5p | SETD5 | 1 | 1 | 2 |
| 65 | hsa-miR-323b-5p | TMEM245 | 1 | 1 | 2 |
| 66 | hsa-miR-323b-5p | HLA-DPB1 | 1 | 1 | 2 |
| 67 | hsa-miR-323b-5p | SPEN | 1 | 1 | 2 |
| 68 | hsa-miR-323b-5p | VASH2 | 1 | 1 | 2 |
| 69 | hsa-miR-323b-5p | TRIP11 | 1 | 1 | 2 |
| 70 | hsa-miR-323b-5p | ST3GAL6 | 1 | 1 | 2 |
| 71 | hsa-miR-323b-5p | FMN2 | 1 | 1 | 2 |
| 72 | hsa-miR-323b-5p | CEBPG | 1 | 1 | 2 |
| 73 | hsa-miR-323b-5p | CDKN2B | 1 | 1 | 2 |
| 74 | hsa-miR-323b-5p | ITGA6 | 1 | 1 | 2 |
| 75 | hsa-miR-323b-5p | TMEM26 | 1 | 1 | 2 |
| 76 | hsa-miR-323b-5p | HOMEZ | 1 | 1 | 2 |
| 77 | hsa-miR-323b-5p | PRICKLE2 | 1 | 1 | 2 |
| 78 | hsa-miR-323b-5p | TRPS1 | 1 | 1 | 2 |
| 79 | hsa-miR-323b-5p | MXD1 | 1 | 1 | 2 |
| 80 | hsa-miR-323b-5p | CLASP2 | 1 | 1 | 2 |
| 81 | hsa-miR-323b-5p | GGA2 | 1 | 1 | 2 |
| 82 | hsa-miR-323b-5p | GTPBP10 | 1 | 1 | 2 |
| 83 | hsa-miR-323b-5p | IPMK | 1 | 1 | 2 |
| 84 | hsa-miR-323b-5p | ALG2 | 1 | 1 | 2 |
| 85 | hsa-miR-323b-5p | ARHGAP29 | 1 | 1 | 2 |
| 86 | hsa-miR-323b-5p | COPS2 | 1 | 1 | 2 |
| 87 | hsa-miR-323b-5p | RANBP6 | 1 | 1 | 2 |
| 88 | hsa-miR-323b-5p | ATP6V1G1 | 1 | 1 | 2 |
| 89 | hsa-miR-323b-5p | ALG13 | 1 | 1 | 2 |
| 90 | hsa-miR-323b-5p | C6orf25 | 1 | 1 | 2 |
| 91 | hsa-miR-323b-5p | HSD17B11 | 1 | 1 | 2 |
| 92 | hsa-miR-323b-5p | ZFYVE16 | 1 | 1 | 2 |
